# Physiologically grounded metrics of model skill: a case study estimating heat stress in intertidal populations

**DOI:** 10.1093/conphys/cow038

**Published:** 2016-10-04

**Authors:** Nicole E. Kish, Brian Helmuth, David S. Wethey

**Affiliations:** 1 Marine Science Program, University of South Carolina, Columbia, SC 29208, USA; 2 Marine Science Center, Northeastern University, Nahant, MA 01908, USA

**Keywords:** Biogeography, climate change, ecological forecasting, intertidal zone, model verification, thermal stress

## Abstract

Models of ecological responses to climate change fundamentally assume that predictor variables, which are often measured at large scales, are to some degree diagnostic of the smaller-scale biological processes that ultimately drive patterns of abundance and distribution. Given that organisms respond physiologically to stressors, such as temperature, in highly non-linear ways, small modelling errors in predictor variables can potentially result in failures to predict mortality or severe stress, especially if an organism exists near its physiological limits. As a result, a central challenge facing ecologists, particularly those attempting to forecast future responses to environmental change, is how to develop metrics of forecast model skill (the ability of a model to predict defined events) that are biologically meaningful and reflective of underlying processes. We quantified the skill of four simple models of body temperature (a primary determinant of physiological stress) of an intertidal mussel, *Mytilus californianus*, using common metrics of model performance, such as root mean square error, as well as forecast verification skill scores developed by the meteorological community. We used a physiologically grounded framework to assess each model's ability to predict optimal, sub-optimal, sub-lethal and lethal physiological responses. Models diverged in their ability to predict different levels of physiological stress when evaluated using skill scores, even though common metrics, such as root mean square error, indicated similar accuracy overall. Results from this study emphasize the importance of grounding assessments of model skill in the context of an organism's physiology and, especially, of considering the implications of false-positive and false-negative errors when forecasting the ecological effects of environmental change.

## Abstract (precis)

Models of biological responses to climate change often use environmental variables measured at large scales, which are only indirect drivers of organism physiology and survival. We used skill scores based on physiology to assess the performance of several biophysical models. While all models had similar levels of overall accuracy using more traditional model skill metrics, their ability to predict stress levels differed.

## Introduction

Accurately predicting the effects of a changing climate on organismal biology is imperative to understanding future species distributions, patterns of biodiversity and changes in ecosystem function. To this end, many studies have extrapolated from physiological tolerances measured in controlled laboratory conditions to the field using readily available environmental data (e.g. Pörtner, 2002; Chown *et al*., 2004; Somero, 2005) that, in some cases, are available only at coarse spatial and/or temporal scales. Such ‘macrophysiological’ approaches (Chown *et al*., 2004) have provided crucial links between physiological and biogeographical responses to environmental change, and offer a potential solution to concerns over model stationarity and assumptions of space-for-time substitution inherent in non-mechanistic models (Stenseth *et al*., 2003). However, problems arise when the temporal and spatial scales of the environmental data being used are not appropriate to the physiological process being investigated. Some data sources (e.g. Worldclim: Hijmans *et al*., 2005) collapse environmental variability into temporal averages, such as monthly means, or else report only habitat-level variables (Kearney, 2006), such as air or land surface temperature. Although such temporally smoothed data are appropriate for other applications, such as climatology, at a proximal level they may have little to do with body temperatures that drive organism performance (Hallett *et al*., 2004; Helmuth *et al*., 2010, 2014; Kearney *et al*., 2012). For example, the body temperatures of ectothermic organisms exposed to direct sunlight can vary substantially from the temperature of the surrounding air or surface (Marshall *et al*., 2010; Wilson and Jetz, 2015) and can fluctuate by 20°C or more over a 24 h period. The use of spatial averages also neglects the large influence of microhabitats, which can potentially provide refugia even in extreme weather conditions (Seabra *et al*., 2011; Suggitt *et al*., 2011; Potter *et al*., 2013). Nevertheless, many studies still rely on single environmental parameters, quite often air, land surface or sea surface temperature, as proxies for organism temperature in the field. More nuanced approaches have been introduced to explore other aspects of these ‘environmental signals’ (Helmuth *et al*., 2010), such as temporal rates of change or climate velocities (Loarie *et al*., 2009; Burrows *et al*., 2011; Lima and Wethey, 2012; Pinsky *et al*., 2013) and return times of sub-lethal extremes (Kearney *et al*., 2012). However, these too are often based on changes in ‘habitat-level’ parameters (Kearney, 2006) and do not account for the highly non-linear mechanisms by which environmental conditions are translated into physiological stress (Kearney *et al*., 2008).

To circumvent these issues, biomimetic sensors have been used to measure animal temperatures directly (Lima *et al*., 2011), and heat budget models have been used to predict organism temperatures, using as inputs multiple climatic parameters, such as air and water temperature, wind speed and solar radiation (Porter and Gates, 1969; Helmuth, 1998; Kearney *et al*., 2010; Wethey *et al*., 2011a). These methods are used to build mechanistic niche models, but they rely on species trait information, such as thermal performance curves, that can be difficult to obtain (Kearney *et al*., 2010; Wethey *et al*., 2011a; Woodin *et al*., 2013) and are therefore very data and time intensive, especially when compared with correlative niche models that can take advantage of large environmental databases. In the few instances when results from mechanistic and correlative approaches have been compared, it is not always clear whether the use of more detailed input data leads to substantially different predictions and whether they are more accurate. Buckley *et al*. (2010), for example, showed that mechanistic and correlative approaches both predicted similar contemporary range boundaries, but that mechanistic approaches predicted larger range shifts under future climate scenarios. Phillips *et al*. (2008) found that correlative models were generally unsuitable for predicting the spread of an invasive cane toad and argued that mechanistic approaches were more reliable. In an effort to circumvent such problems, some modellers have begun to include bioclimatic indicators that are thought to be more directly related to a species’ biology; for example, seasonal extremes (Briscoe *et al*., 2016). Nevertheless, the inclusion of these metrics does not necessarily improve model performance (e.g. Watling *et al*., 2012; Bucklin *et al*., 2014; but see Briscoe *et al*., 2016). The question of how much ‘detail’ needs to be included in modelling frameworks to predict changes over large geographical scales successfully therefore remains unresolved.

A central problem facing climate scientists is thus how to quantify model skill, i.e. the ability of a model to predict a series of defined events, such as exceedance of some critical threshold. This problem is especially acute when forecasting future responses in what will probably be novel conditions, and to decide how much detail and what variables are needed in order to produce useful predictions for any given application (Brown *et al*., 2011; Helmuth *et al*., 2014; Briscoe *et al*., 2016; Olsen *et al*., 2016). A key assumption in modelling the responses of populations and species to environmental change is that the metric(s) being used to test model performance is biologically relevant (Stenseth *et al*., 2003; Hallett *et al*., 2004; Seebacher and Franklin, 2012). For example, Woodin *et al*. (2013) found that extreme (lethal) temperatures appeared to have high explanatory power for describing species distributions of mussels in North America, but had virtually no predictive value when applied to the same species on the coast of Europe. Instead, distributions were best explained by repeated exposure to chronic physiologically stressful conditions, and thus required the use of an energetics model. This result is likely to have occurred because on the East coast of North America multiple drivers of lethality (extremes in temperature and sub-lethal conditions that cause energetic failure) covary spatially with one another. As a result, on this coast animals living at their geographical limits died from heat extremes before they had time to run out of energy. In contrast, on the coast of Europe, where physiological energetics and thermal mortality risk are decoupled in space, only energetics were useful in describing shifts in range boundaries.

In other words, given that there are multiple factors that can cause reproductive failure and mortality, and these may not always coincide in space and/or time (Hallett *et al*., 2004), it is often necessary to quantify multiple layers of ‘physiological stress’ when mapping the response of organisms to environmental change. Here, we define ‘stress’ as any physiological response that reduces fitness or otherwise deviates from optimal functioning, including non-lethal consequences, such as reductions in growth and reproduction. Comparatively few methods for forecasting the impacts of environmental change on species distribution patterns take such an approach, however, and it is still far more common to correlate presence/absence directly with environmental data. As a consequence, it is not always easy to determine whether particular environmental variables or commonly used metrics, such as seasonal averages or even monthly extremes, hold true explanatory power *per se* or whether they are simply well correlated with metrics that genuinely affect organismal performance (Stenseth *et al*., 2003). Such discussions are at the heart of concerns over model stationarity and space-for-time substitution, as existing correlations among climatic variables, or even among different measures of the same variable (e.g. means and extremes), may not necessarily hold true in the future (Milly *et al*., 2008). Thus, mechanistic models that use weather data collapsed into monthly averages may appear to have similar skill to correlative approaches (Buckley *et al*., 2010), but this may be because in both models the environmental metrics being used as the input variables (monthly means) fail to account for the role of high-frequency variation in factors such as body temperature (Kearney *et al*., 2012; Montalto *et al*., 2014) or the temporal coincidence between factors such as thermal and hydric stresses (Hallett *et al*., 2004; Briscoe *et al*., 2016).

Comparable arguments have been made for why climatic indices, such as the El Niño Southern Oscillation, appear to have higher predictive power, even though at the scale of organisms these indices have little direct biological meaning; an individual organism cares little for what the El Niño Southern Oscillation index is, but it does care about patterns of temperature, rainfall and food supply in its immediate vicinity that are correlated with the index (Stenseth *et al*., 2003). Hallett *et al*. (2004), studied populations of sheep and found that, at least superficially, the North Atlantic Oscillation displayed better correlations with population dynamics than did local weather patterns. The cause of this rather enigmatic result was made clear only when details regarding the roles of factors such as the history of extreme events, energetic state and food availability were included in a more process-based model. Using this latter approach, weather emerged as a stronger predictor of the sheep's population patterns than did the North Atlantic Oscillation.

Here, we address the question of how much detail is needed in order to make effective predictions of animal body temperature and, hence, physiological stress for any particular application, or when less time- and data-intensive approaches can give sufficient insight. Specifically, this study asks, is it always necessary to construct a full heat budget model when investigating the biological impacts of changing air temperatures or can simple metrics, such as air temperature, in fact be informative of relative physiological stress, even though we know that strictly speaking they may be only marginally connected to body temperature and thus to physiological responses? Likewise, how well can simple correlations based not only on air temperature but also on solar radiation predict physiological stress (Elvin and Gonor, 1979; Wilson and Jetz, 2015)?

**Figure 1:**
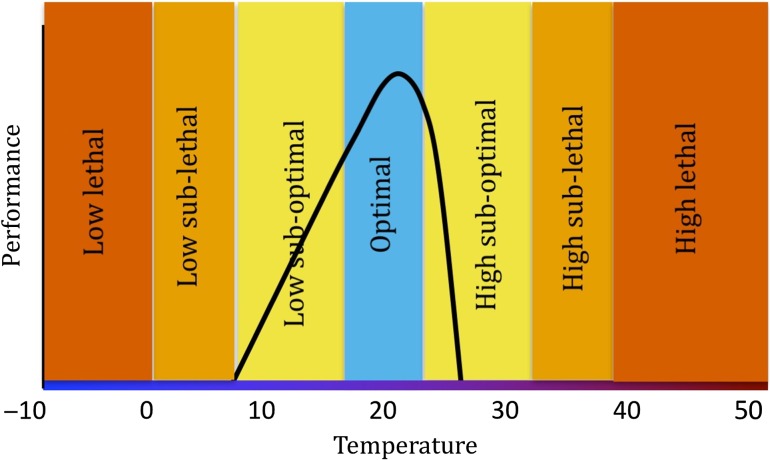
Typical thermal performance curve (TPC). A TPC describes the relationship between body temperature and some metric of physiological performance relevant to fitness; for example, feeding rate. Thermal performance curves are often left (negative)-skewed so that small changes in body temperature above optimal levels can lead to large decreases in performance. Based on physiological data, we divided the TPC for mussels into seven performance categories, which were used to test model skill.

We couch this question in the context of the thermal sensitivity of organismal physiology, as typically measured by a thermal performance curve (TPC; Fig. 1). There is a long-standing history of using TPCs to describe the general relationship between an organism's body temperature and factors related to fitness, such as growth or feeding rate (Dell *et al*., 2013), although recent authors have highlighted several limitations (Kingsolver and Woods, 2016). For example, for an organism with a broad thermal performance width, a difference of several degrees may matter little for predictions of physiological performance over a large part of its TPC, especially at temperatures below optimum (Fig. 1). In stark contrast, when body temperatures are close to lethality or metabolic failure, an error of a few degrees may make the difference between predicting whether the animal will live or die (Ruel and Ayres, 1999).

Here, we compare the predictive capacity of five methods for quantifying body temperature (and, thus, thermal performance) of the ecologically dominant intertidal California mussel, *Mytilus californianus*, using a physiological framework. *In situ* body temperatures of living mussels were collected by biomimetic sensors deployed over a period of 7–11 years at five sites spanning 10° of latitude along the west coast of the USA (Helmuth *et al*., 2006). Using a thermal physiology framework, we divided both measured and predicted temperatures into physiologically based categories of optimal, sub-optimal (in terms of both heat and cold stress), sub-lethal and lethal exposure (Fig. 1). We analysed the overall accuracy of each method in predicting body temperatures using common statistical metrics, such as root mean squared error (RMSE), but in addition (and in contrast to previous studies) we also compared the skill of each method in accurately capturing categories of physiological performance experienced by mussels. We also applied several forecast verification techniques used in meteorological research to predictions made by the models. These approaches allow the assessment of a model's ability to predict the incidence of multiple levels of physiological stress, rather than simply mean conditions.

Forecast verification techniques have received considerable attention in meteorological research, because an unskilled weather forecast can compromise public safety and result in economic loss (Thornes and Stephenson, 2001). As a result, a variety of verification metrics and skill scores have been designed to assess different aspects of forecast quality; these skill scores reflect the overall association between forecasts and observations and measure how much more skilful a forecast is than some reference (commonly used baselines include climatology, persistence and chance; Stephenson, 2000; Thornes and Stephenson, 2001). Owing to the potential of rare extreme weather events to cause significant societal impacts and losses and the predicted increase in the frequency of these events resulting from anthropogenic climate change (Kodra and Ganguly, 2014), the interest in verification methods for extreme events has grown in recent years (Casati *et al*., 2008). Here, we test the skill of multiple methods of estimating body temperature, using metrics of physiological performance and stress as indicator categories (Fig. 1).

## Materials and methods

### Study sites and organisms

The rocky intertidal zone is a stressful, constantly changing environment, in which organisms must contend alternately with terrestrial and marine conditions on sub-daily time scales. Body temperatures of organisms in this habitat are driven by multiple aspects of the environment, such as air temperature, water temperature, solar irradiance, wave splash, wind speed and the timing of the tides (Helmuth, 1998, 1999, 2002; Denny and Harley, 2006; Wethey *et al*., 2011a). Body temperatures of intertidal organisms often differ greatly from the temperature of the ambient air, water or surface (e.g. Southward, 1958; Lewis, 1963; Etter, 1988), and some aspect of thermal stress is thought to be among the major determinants of distribution on local and geographical scales (Southward, 1958; Wethey, 1983; Somero, 2002). Recent work has also emphasized, however, that for some species lethally high temperatures are likely to be a poor predictor of local distribution in many sites (Mislan *et al*., 2014) and that, like the aforementioned mussels in Europe (Woodin *et al*., 2013; Fly *et al*., 2015; Seabra *et al*., 2015), other aspects of thermal stress are also important.

Given that their survival and physiological performance are so closely tied to aspects of the physical environment, sessile intertidal ectotherms may be especially vulnerable to the changes brought about by climate change and can serve as an effective tool for estimating its effects (Denny and Harley, 2006; Wethey *et al*., 2011a). The mussel *M. californianus* is one of the most ecologically important species on rocky shores of the west coast of North America. As an engineering species, it creates habitat for small fauna (Suchanek, 1979), while at the same time competitively excluding other larger organisms (Paine, 1974). Although juvenile mussels are somewhat mobile (Harger, 1968; Schneider *et al*., 2005), adults are almost entirely sessile and have no ability to relocate in response to thermal stress. Several biophysical models have been developed to estimate mussel body temperatures (Helmuth, 1998; Gilman *et al*., 2006b; Helmuth *et al*., 2011; Wethey *et al*., 2011a), and for well over a decade our research group has maintained a database of *in situ* temperatures at multiple sites using biomimetic data loggers (Helmuth *et al*., 2002; Helmuth *et al*., 2006). These instruments match the thermal characteristics of living mussels to within ~2°C and have been proved to serve as a highly effective means of quantifying mussel temperature in the field (Fitzhenry *et al*., 2004).

### *In situ* temperature measurements

**Figure 2:**
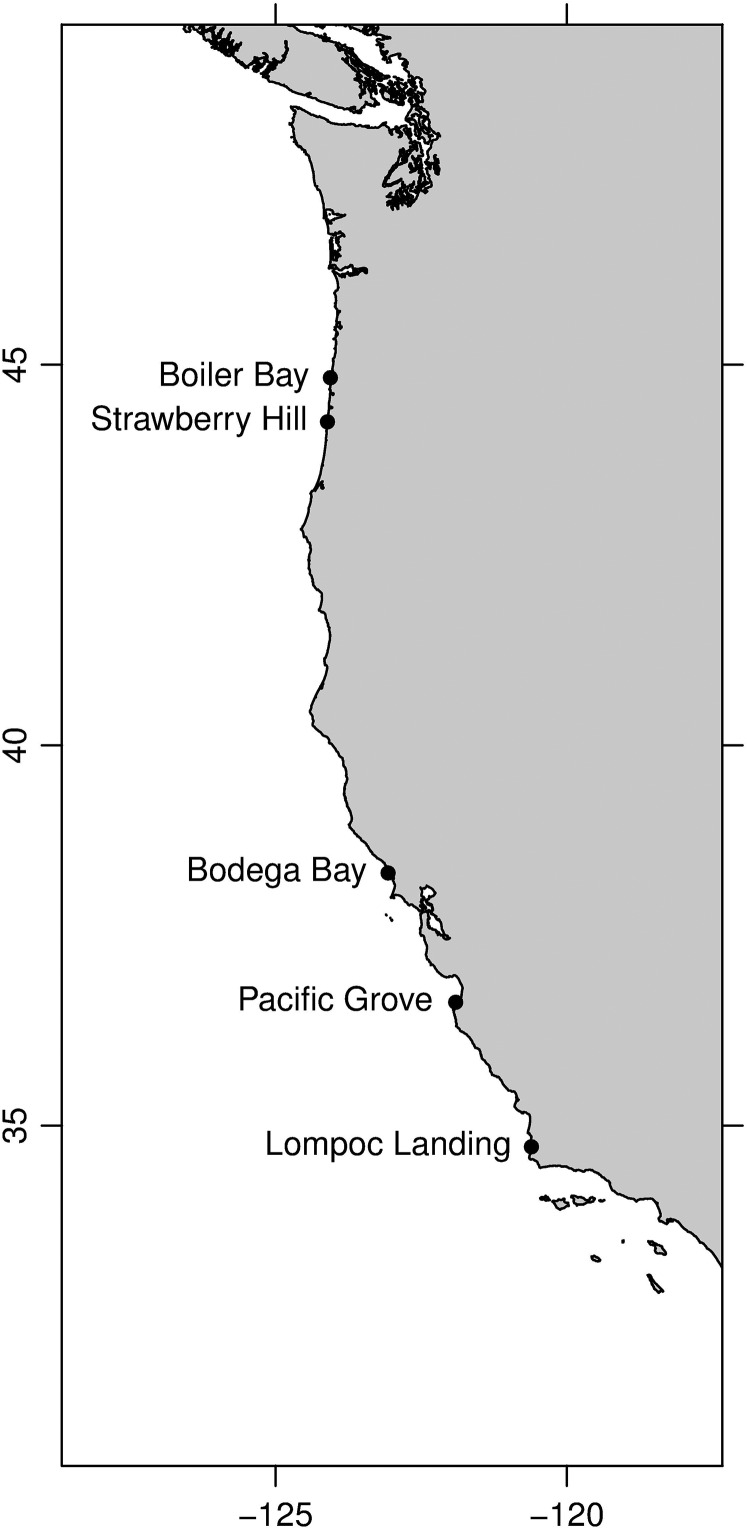
Location of study sites along the west coast of the USA. Biomimic loggers were deployed at five sites spanning ~10° of latitude in Oregon and Washington. See Table 1 for deployment dates.

Data were collected using biomimetic sensors from five sites over a period spanning 7–11 years (see Table 1 for site co-ordinates and dates of deployment) along the west coast of the USA (Fig. 2; Boiler Bay and Strawberry Hill, OR, USA; and Bodega Bay, Pacific Grove and Lompoc Landing, CA, USA) using methods described by Helmuth *et al*. (2006). At each site, ‘robomussels’, biomimetic sensors designed to replicate the thermal characteristics of mid-size (~75 mm shell length) *M. californianus* mussels (Fitzhenry *et al*., 2004), were deployed in growth position in mussel beds in the mid-intertidal zone (mean lower low water +1.5 m). Robomussels consisted of a Tidbit datalogger (Onset Computer Corporation) encased in polyester resin (Fitzhenry *et al*., 2004). Given that the local topography and substratum angle can result in significant differences in the body temperature of individuals (e.g. Helmuth and Hofmann, 2001; Denny *et al*., 2011; Seabra *et al*., 2011), only loggers deployed on horizontal surfaces on wave-exposed benches at mid-tidal elevations were considered in this analysis. Loggers were programmed to collect data every 10 min and deployed for ~6 months, at which point they were collected and replaced with new loggers. Five loggers were deployed at each site; however, as a result of wave action, some instrument loss occurred. On average, three loggers were recovered at each time of instrument retrieval. Brief gaps (~1–2 days) in the data record occurred when loggers were replaced.

**Table 1:** Dates of logger deployment at each site

Site	Longitude	Latitude	Deployment dates	Number of loggers retrieved
Boiler Bay	−124.06	44.83	8 April 2002–16 May 2009	3
Strawberry Hill	−124.11	44.25	22 May 2000–23 May 2001	2–4
			9 April 2002–7 October 2002	
			20 Apr 2003–4 May 2004	
			29 August 2004–10 June 2010	
Bodega Bay	−123.07	38.32	8 July 2004–8 March 2012	3
Pacific Grove	−121.91	36.62	30 June 2000–6 Dec 2009	4
			3 July 2011–23 April 2012	
Lompoc	−120.61	34.72	9 February 2002–3 November 2009	2

### Environmental data

Weather station data (air temperature, wind speed and significant wave height) were obtained from the nearest National Buoy Data Center buoy (http://www.ndbc.noaa.gov/), with two exceptions. Hourly average air temperature and wind speed data for Bodega Bay were obtained from the Bodega Ocean Observing Node (http://bml.ucdavis.edu/boon/), and 10 min air temperature and wind speed data for Pacific Grove were obtained from a local weather station maintained by the Hopkins Marine Station Marine Life Observatory (http://mlo.stanford.edu/) and located immediately adjacent to the logger deployment site. Data from Pacific Grove were averaged over the course of each hour to allow their rectification with buoy data. At certain times, National Buoy Data Center data were available only as point measurements on the hour; given that air and water temperature change relatively slowly, during these intervals each observation was averaged with the following observation to obtain an hourly mean.

Direct solar radiation is not frequently measured by weather stations. Local solar radiation data were available at Pacific Grove for the entire period of record (M. W. Denny, http://mlo.stanford.edu/) and at Bodega Bay beginning in August 2008. For all other sites, gridded direct solar radiation data were obtained from the GEWEX Continental Scale International Project (GCIP; Pinker and Lazlo, 1992) Surface Radiation Budget (http://www.atmos.umd.edu/~srb/gcip/). Surface downwelling shortwave hourly average data are available from a ½ degree grid for the years 1996–2010; the data were closely correlated with local solar radiation measurements from Pacific Grove (Pearson correlation 0.909, *P* < 0.01). When included in a biophysical model, results using GCIP solar data as inputs have been shown to be similar to results using local weather station data (Mislan and Wethey, 2011). The GCIP data set is limited in that data are available only between the hours of 09.00 and 16.00 h local standard time, so apparent solar azimuth and elevation for each site were obtained from the NASA Jet Propulsion Laboratory HORIZON's Web interface (http://ssd.jpl.nasa.gov/horizons.cgi). Solar radiation observations were assumed to be zero at times when apparent solar elevation was negative; the remaining missing observations were obtained by linearly interpolating from sunrise until 09.00 h local time or from 16.00 h local time until sunset.

Given that *M. californianus* experiences its most thermally stressful conditions during periods of aerial exposure, only data taken when mussels were exposed at low tide were considered in this analysis. For each study site, observed tidal height data were obtained from the nearest National Oceanic and Atmospheric Administration (NOAA) Center for Operational Oceanographic Products and Services observing station (http://tidesandcurrents.noaa.gov/). The effect of wave splash was estimated using metrics of significant wave height and shore slope data presented by Gilman *et al*. (2006a). Wave run-up (swash) for each site was added to measurements of still tidal height; these data were used to separate periods of emersion and immersion based on the absolute tidal height of the loggers.

### Modelling approaches

We took five different approaches to modelling the body temperatures of *M. californianus*. The first of these was to use the air temperature at low tide as a proxy for body temperature. It has been shown that the body temperatures of many ectothermic species are strongly affected by solar radiation (Marshall *et al*., 2010) and that the body temperature of organisms frequently differs—often greatly—from the ambient air temperature (e.g. Southward, 1958; Helmuth, 1998; Kearney, 2006; Chapperon and Seuront, 2011). Nevertheless, air temperature is still frequently used in climate envelope studies of species distributions, as well as in some physiological studies (Helmuth *et al*., 2014). In addition, there is evidence to suggest that air temperature may, in some cases, serve as an indirect proxy of body temperature. On cloudy days in the absence of cooling from evaporation, air temperature generally sets the lower limit of body temperature, and solar radiative heat gain then increases organism temperature above that minimum (Helmuth, 1998). Air temperature may therefore be informative when one is modelling the distribution of refuges for populations, which occur in fully shaded microhabitats (e.g. Jones *et al*., 2010; Lima *et al*., 2016). The reverse occurs during cloud-free days at night, when body temperatures dip several degrees below local air temperature because of heat loss via long-wave radiation. Air temperature may be useful as a proxy when cold stress events are of interest (e.g. Firth *et al*., 2011), although the degree of correspondence will vary with cloud cover. Mislan *et al*. (2014) recently showed that sites where intertidal mussels were likely to experience lethal extremes were characterized by the highest air temperatures; they also, however, showed that full exposure to solar radiation was required for these temperatures to be achieved.

The second approach was a simple regression model first presented by Elvin and Gonor (1979). Between 1970 and 1974, Elvin and Gonor collected data on *M. californianus* tissue temperatures, solar radiation, water temperature, air temperature, wind speed and relative humidity at Yaquina Head, OR, USA, located ~20 km from Boiler Bay and ~45 km from Strawberry Hill. Their aim was to estimate the thermal regime for *M. californianus* to determine scope for growth. From these data, they calculated that hourly tissue temperatures could be estimated by the formula:

(1a) $$T_{\mathrm{mussel}}\;=\;T_{\mathrm{air}}\;+\;5.03\;\times \;L$$

where *L* represents solar isolation in Langleys per minute, and *T*_air_ and *T*_mussel_ represent air temperature and mussel body temperature (in degrees Celsius), respectively. We converted the equation to units of the more commonly used watts per square metre; 1 W/m^2^ = 1.43 × 10^−3^ Langleys min^−1^:

(1b) $$T_{\mathrm{mussel}}\;=\;T_{air}\;+\;7.21\;\times 10^{-3}\;\times \;S$$

where *S* is direct solar radiation in units of watts per square metre. Thus, for example, at a relatively high *S* value of 1000, mussel body temperature would be estimated as ~7°C warmer than air temperature.

We applied this formula to data collected at Boiler Bay and Strawberry Hill as well as our study sites in California; hereafter, we refer to it as the Elvin and Gonor model.

We also modified the Elvin and Gonor model at each site to test the concept of model stationarity, i.e. that the relationship between environmental variables (air temperature and solar radiation) and body temperature would remain constant across sites. For each study site, we performed multiple regressions of air temperature and solar irradiance against robomussel temperature. Although use of a multiple regression introduces error into the predictions (because correlation exists between air temperature and solar radiation), this method is commonly used in situations such as the Elvin and Gonor model or in the prediction of sea turtle nest temperatures (e.g. Hawkes *et al*., 2007). It also presents a potentially more tractable approach that does not require the application of a heat budget model, although extensive field data are necessary.

The fourth approach was a simplified steady-state biophysical model, presented by Helmuth *et al*. (2011) and based on a model first presented by Helmuth (1999) and modified by Kearney *et al*. (2010). This generic model, developed and tested for loggers at Bodega Bay, is designed to calculate hourly body temperatures of mid-size (~75 mm) mussels in a horizontal bed. The model uses hourly measurements of solar irradiance, air temperature and wind speed as inputs to estimate heat gain and/or loss resulting from direct short-wave solar radiation, infrared radiation to and from the sky, infrared radiation to and from the ground, conduction to and from the ground, and convection to and from the surrounding air, as modified by the morphological characteristics of the mussels themselves (Helmuth, 1999). For simplicity, the model assumes that ground temperature and air temperature are equivalent, that cloud cover is constant with regard to infrared heat exchange and that the effect of evaporative cooling is negligible (Helmuth *et al*., 2011). This deliberately simplified model also assumes that mussels reach equilibrium within an hour; the ‘thermal inertia’, or the time constant that reflects the ability of an organism to resist changes in temperature, for a mid-size mussel is approximately 20–30 min (Helmuth, 1999; Helmuth *et al*., 2011), so this assumption is reasonable.

The fifth approach was a biophysical model of the time dynamics of body temperature (NOAH mussel model: Wethey *et al*., 2011a). This model uses gridded meteorological data from the North American Regional Reanalysis (Mesinger *et al*., 2006) for additional variables beyond those described in the ‘*Environmental data*’ section, as follows: downwelling long-wave radiation, relative humidity at 2 m, atmospheric pressure and precipitation rate. The NOAH mussel biophysical model was derived from the NOAH (named for its heritage---N: National Centers for Environmental Prediction; O:Oregon State University Dept Atmospheric Sciences; A:Air Force Weather Agency and Air Force Research Lab; H:Hydrologic Lab National Weather Service) land surface model (Ek *et al*., 2003), which is used to predict soil temperatures and hydrology in the NOAA operational forecasting models (Wethey *et al*., 2011a). North American Regional Reanalysis data were available on a spatial grid of ~30 km and at 3 h time intervals, so they were bilinearly interpolated in space to the locations of the robomussel sites and linearly interpolated in time to provide values at 30 min intervals as inputs to the mussel model. The use of gridded North American Regional Reanalysis data has been validated with the mussel model (Mislan and Wethey, 2011). The mussel model has a layer of mussels 8 cm thick on top of granite rock. At low tide, the model simulates radiative, convective, conductive and evaporative heat exchanges, and at high tide, the mussels and uppermost rock surface under the mussel layers return to Sea Surface Temperature (SST), and the lower rock layers change temperatures by conduction. Rock temperature during submersion thus sets the initial conditions of the underlying rock during low tide such that water temperature can indirectly affect the low-tide temperature of mussels (Wethey, 2002). Mussel temperatures were calculated at two depths from the top of the simulated bed, 3 and 5 cm.

For Boiler Bay, Bodega Bay, Pacific Grove and Lompoc Landing, only data collected when all observations were present were included in the analysis, i.e. if data were missing from one microsite or one environmental parameter, observations of other parameters taken at that same time were not included. At Strawberry Hill, three out of any four mid-exposed horizontal microsites had loggers deployed at any particular time throughout the entire period of record. Given that excluding data where observations were missing for one microsite would have limited the data set to only a few hundred observations (when four loggers were deployed at once), we analysed each microsite individually.

A thermal physiology framework based on thermal performance curves was developed to categorize results (Fig. 1). ‘Optimal’ body temperature was defined as 17–22°C, the range at which the highest scope for growth is reported (Bayne *et al*., 1976). Zero scope for growth is believed to occur at ~26°C (Bayne *et al*., 1976); heat shock proteins are observed when body temperatures exceed 32°C; and Denny *et al*. (2011) reported the LT_50_ (median lethal temperature) to be 38.2°C. ‘High sub-optimal’ was therefore defined to range from 22 to 32°C, ‘high sub-lethal’ as 32–38°C, and ‘high lethal’ as temperatures exceeding 38°C. Less work has been done regarding cold stress in *M. californianus*; however, exposure to freezing conditions is known to be lethal (Seed and Suchanek, 1992). Yao and Somero (2012) reported double-stranded DNA breaks and caspase-3 activation, a signal of apoptosis, in *M. californianus* at 2 and 6°C, but not at 13°C, although it is also important to note that the stress response was time dependent. ‘Low sub-optimal’ was defined as 10–17°C and ‘low sub-lethal’ as 0–10°C. Temperatures <0°C were defined as ‘low lethal’. Note that here, as a simplifying assumption, we consider thermal performance curves to be static over time, i.e. that no acclimatization or local adaptation occurred (Kingsolver and Woods, 2016).

### Model skill metrics

Given that forecasting the effects of climate change on ecosystems requires accurate prediction of temperatures along the entire range of the probability distribution as well as mean conditions, we calculated a number of forecast verification statistics used in meteorological research. As these metrics rely on paired data consisting of one forecast value and one observed value (Wilks, 1995), we calculated the daily maximal temperature, defined as the highest hourly average measurement in a 24 h period, for both the forecast and the observed values. The daily maximum was chosen as a metric because it eliminates the need for the models to match the temperature trajectory of the observations while still remaining indicative of physiological stress. In other words, the time of day when a mussel achieved maximal temperature was less important than the temperature it ultimately experienced.

Using daily maximum data, we calculated the bias of an estimator (here defined as the predicted value minus the observed value), mean absolute error (MAE) and root mean squared error (RMSE)—metrics which have commonly been used to evaluate ecological model performance—for each model relative to logger temperature. We also assessed model performance by applying several forecast verification techniques used by the meteorological community to our data. Hit rate and false-alarm ratios were used to assess performance within a particular temperature band, and the Heidke score, Peirce score and Gerrity score to assess overall model performance (Center for Australian Weather and Climate Research, http://www.cawcr.gov.au/projects/verification/). An occurrence where both the predicted and observed temperatures fell within the same range (category) of physiological stress was defined as a ‘hit’. If models predicted temperatures to fall within one range of physiological performance, whereas in reality they fell within another band of physiological stress, this occurrence was defined as a ‘false alarm’ for the predicted range. Given that temperatures fall along a continuum, a ‘false alarm’ within one band is also a ‘miss’ within another.

The hit rate indicates the relative number of times an event that was forecasted occurred, and can be calculated using the following formula:

(2) $$\mathrm{Hit rate}=\frac{\mathrm{Hits}}{\mathrm{Hits}+\mathrm{Misses}}$$

Scores range from 0 to 1, where 0 indicates no skill and 1 indicates a perfect forecast.

The false-alarm ratio indicates the relative number of times an event that was forecast to occur did not:

(3) $$\text{False}-\text{alarm ratio}=\frac{\mathrm{False alarms}}{\mathrm{Hits}+\mathrm{False alarms}}$$

Like the hit rate, scores range from 0 to 1, but here, 1 indicates no skill and 0 a perfect forecast. (Stephenson, 2000).

For multicategory forecasts, the Heidke skill score is calculated using the following equation:

(4) $$\mathrm{Heidke score}\;=\;\frac{\left[\frac{1}{N}\sum_{i=1}^{k}n\left(F_{i}O_{i}\right)-\frac{1}{N^{2}}\sum_{i=1}^{k}n(F_{i})n(O_{i})\right]}{\left[1-\frac{1}{N^{2}}\sum_{i=1}^{k}n\left(F_{i}\right)n(O_{i})\right]}$$

where *n*(*F*_*i*_*O*_*i*_) is the number of forecasts in category *i* that have observations in category *i*, *n*(*F*_*i*_) is the number of forecasts in category *i*, *n*(*O*_*i*_) is the number of observations in category *i*, and *N* is the total number of forecasts. This score compares the proportion of correct forecasts with the proportion correct obtained from a no-skill random forecast (Stephenson, 2000) and relies on the hit rate as the basic measure of accuracy (Wilks, 1995). This score ranges from −1 to 1, where a score of 0 indicates no skill and a score of 1 indicates a perfect forecast. The Heidke skill score is considered a more impartial measure of forecast skill than the proportion correct, because proportion is strongly influenced by the most common category (such as ‘no event’ for rare occurrences) and, as a result, can be misleading (Stephenson, 2000).

The Peirce score (Peirce, 1884) represents the difference between the hit rate and false-alarm rate. It is calculated using the following equation:

(5) $$\mathrm{Peirce score}\;=\;\frac{\left[\frac{1}{N}\sum_{i=1}^{k}n\left(F_{i}O_{i}\right)-\frac{1}{N^{2}}\sum_{i=1}^{k}n(F_{i})n(O_{i})\right]}{\left[1-\frac{1}{N^{2}}\sum_{i=1}^{k}{[n(O_{i})]}^{2}\right]}$$

When the score is >0, the number of hits exceeds the number of false alarms. Like the Heidke score, the Peirce score ranges from −1 to 1, with 0 indicating no skill and 1 indicating a perfect forecast.

The Gerrity score (Gerrity, 1992) is calculated using the following equation:

(6a) $$\mathrm{Gerrity score}\;=\;\frac{1}{N}\sum_{i=1}^{k}\sum_{j=1}^{k}n(F_{i}O_{j})s_{\mathrm{ij}}$$

where *s*_*ij*_ are elements of a scoring matrix. On the diagonal, (*i* = *j*), *s*_*ij*_ is given by:

(6b) $$s_{\mathrm{ij}}=\frac{1}{k-1}\;*\left(\sum_{r-1}^{i-1}{a_{r}}^{-1}+\sum_{r=1}^{k-1}a_{r}\right)$$

and off-diagonal (*I ≠ j*), *s*_*ij*_ is given by:

(6c) $$s_{\mathrm{ij}}=\frac{1}{k-1}\;*\;\left(\sum_{r-1}^{i-1}{a_{r}}^{-1}-\left(j-i\right)+\sum_{r=i}^{k-1}a_{r}\right)$$

where:

(6d) $$a_{i}=\frac{1-\sum_{r=1}^{i}p_{r}}{\sum_{r=1}^{i}p_{r}}$$

and

(6e) $$p_{i}=\frac{n(O_{i})}{N}$$

Like the Peirce and Heidke scores, the Gerrity score evaluates the skill of a forecast in predicting the correct category relative to random chance. The Gerrity score likewise ranges from −1 to 1, where a score of 0 indicates no predictive skill and a score of 1 represents a perfect forecast.

Hit rate, false-alarm rate and the Peirce, Heidke and Gerrity scores were calculated using the ‘verification’ package (NCAR-Research Application Program, 2012) in the R statistical software system (cran.r-project.org). As a result of the high within-site variability of temperatures in the intertidal zone (Denny *et al*., 2011), bias, RMSE, MAE and forecast verification metrics were calculated using daily maximal temperatures from each individual microsite as a separate set of observations. The result is a range of statistics that may be indicative of variability in the field.

In order to quantify the effect of variability further, verification statistics were compared among replicate measurements on the ground. That is, data collected by one logger at a particular site were considered a ‘forecast’ and data from another ‘observations’; hit rate and false-alarm ratios were calculated accordingly. If verification statistics calculated using a model as a forecast were similar to those calculated for the interlogger comparison, that model could be considered at least as useful as deploying an additional logger in the field. The results from the interlogger analysis are displayed alongside the results from each model forecast.

## Results

With the exception of air temperature, for which errors were substantially larger than for all other metrics examined, bias, MAE and RMSE reflected relatively little difference in model skill among the biophysical and regression approaches when applied to the same site (Table 2). These methods also indicated larger errors at the northernmost (Boiler Bay) and southernmost (Lompoc Landing) sites (Table 2).

**Table 2:** Bias, root mean squared error and mean absolute error of all models at all sites, for all physiological categories

Parameter	Boiler Bay	Strawberry Hill	Bodega Bay	Pacific Grove	Lompoc	Average	SD
Bias							
Air temperature	−4.83	−4.44	−2.93	−3.56	−6.45	−4.44	1.34
Elvin and Gonor model	−2.51	−1.02	0.93	−0.34	−2.66	−1.12	1.51
Multiple regression	−1.53	−0.84	−0.91	−0.86	1.41	−0.55	1.13
Biophysical model	−2.21	−0.76	1.95	0.69	−2.78	−0.62	1.97
NOAH 3 cm	−0.21	−0.52	0.91	−0.04	−0.92		
NOAH 5 cm	−3.04	−2.19	0.73	−1.58	−2.75		
Among loggers	1.6	1.27	−1.81	−1.07	−0.21		
Root mean squared error							
Air temperature	8.17	5.55	5.25	6.11	8.83	6.78	1.62
Elvin and Gonor model	5.9	4.07	4.2	4.13	5.45	4.75	0.86
Multiple regression	4.8	3.91	4.29	4.17	4.52	4.34	0.34
Biophysical model	5.88	4.03	4.73	4.39	5.57	4.92	0.78
NOAH 3 cm	4.58	4.32	5.25	4.23	4.09		
NOAH 5 cm	5.7	4.55	4.22	4.41	5.04		
Among loggers	4.23	4.27	3.87	3.35	3.14		
Mean absolute error							
Air temperature	5.62	3.72	3.68	4.51	6.88	4.88	1.37
Elvin and Gonor model	3.96	2.7	3.26	3.08	3.85	3.37	0.53
Multiple regression	3.31	2.6	3.08	3.1	3.53	3.12	0.34
Biophysical model	3.99	2.72	3.73	3.29	4.01	3.55	0.55
NOAH 3 cm	3.18	3.07	3.61	3.17	3.1		
NOAH 5 cm	3.92	3.18	3.34	3.3	3.14		
Among loggers	2.86	3.04	2.87	2.36	2.15		

Bias, MAE and RMSE are all measures of average error between a prediction and a model. As sample sizes are small near the tails of a probability distribution, errors in this region would need to be very large to outweigh small errors nearer to the mean to affect overall model accuracy. Although these metrics indicated relatively little difference among models (Table 2), forecast verification techniques indicated larger differences in model skill and provide a more detailed understanding of the strengths and weaknesses of each approach (Table 3). Peirce and Heidke scores for the use of air temperature as a proxy were poor overall, ranging from 0.02 to 0.23 and 0.02 to 0.26, respectively. At Bodega Bay, Pacific Grove and Lompoc Landing, all three scores of air temperature indicated very little improvement in skill vs. a random forecast, although they indicated a slightly larger improvement in skill at Boiler Bay and Strawberry Hill (Table 3). Still, scores for air temperature at the northern sites were poorer than for other methods at the same locations, which was probably a result of the strong role of solar radiation in driving body temperatures often well above air temperature at these sites (Helmuth *et al*., 2010). This strongly cautions against the use of air temperature alone as a proxy, especially when heat stress is of concern. It is also important to note the interlogger variability in skill scores, especially considering that each set of observations was taken by instruments separated by tens of metres and deployed at similar heights and surface orientations (Denny *et al*., 2011).

**Table 3:** Forecast verification skill scores for all models at all sites, for all physiological categories

Score	Boiler Bay	Strawberry Hill	Bodega Bay	Pacific Grove	Lompoc	Average	SD
Air temperature							
Peirce	0.14	0.23	0.07	0.09	0.02	0.11	0.08
Heidke	0.16	0.26	0.08	0.1	0.02	0.12	0.09
Gerrity	NA	NA	0.22	0.05	0.07	0.11	0.09
Elvin and Gonor model							
Peirce	0.29	0.37	0.3	0.33	0.24	0.31	0.05
Heidke	0.31	0.4	0.26	0.32	0.25	0.31	0.06
Gerrity	NA	NA	0.3	0.23	0.19	0.24	0.06
Simple biophysical model							
Peirce	0.3	0.37	0.29	0.36	0.24	0.31	0.05
Heidke	0.32	0.4	0.24	0.34	0.25	0.31	0.07
Gerrity	NA	NA	0.37	0.28	0.2	0.28	0.09
Multiple regression							
Peirce	0.42	0.41	0.23	0.32	0.35	0.34	0.08
Heidke	0.41	0.42	0.23	0.31	0.35	0.34	0.08
Gerrity	NA	NA	0.27	0.17	0.27	0.24	0.06
NOAH 3 cm layer							
Peirce	0.38	0.34	0.32	0.42	0.36	0.36	0.07
Heidke	0.35	0.31	0.29	0.38	0.36	0.33	0.07
Gerrity	0.37	0.19	0.2	0.51	0.32	0.38	0.09
NOAH 5 cm layer							
Peirce	0.15	0.18	0.34	0.44	0.28	0.3	0.12
Heidke	0.18	0.19	0.29	0.43	0.29	0.29	0.11
Gerrity	0.1	0.06	0.16	0.4	0.26	0.27	0.11
Among loggers							
Peirce	0.52	0.43	0.38	0.51	0.56	0.46	0.07
Heidke	0.4	0.36	0.4	0.55	0.56	0.46	0.09
Gerrity	0.36	0.27	0.19	0.51	0.62	0.51	0.11

**Figure 3:**
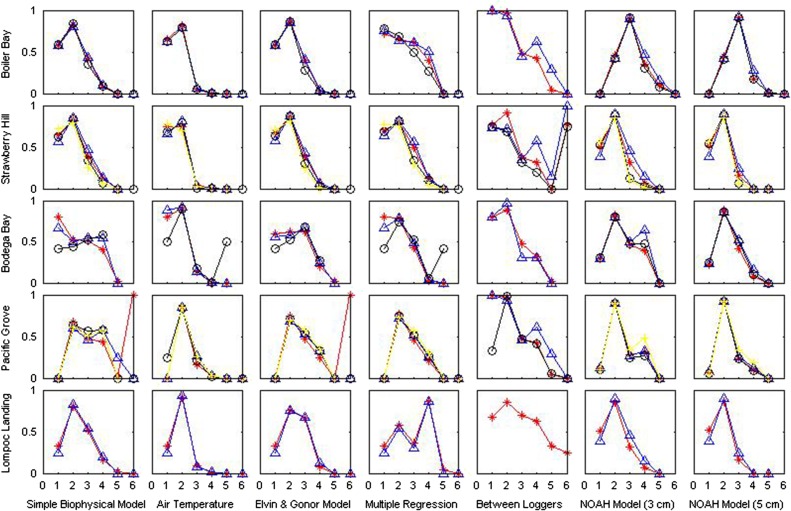
Hit rates for all models at all sites. Categories are as follows: 0 = body temperatures <0°C (low lethal); 1 = 0–10°C (low sub-lethal); 2 = 10–17°C (low sub-optimal); 3 = 17–22°C (optimal); 4 = 22–32°C (high sub-optimal); 5 = 32–38°C (high sub-lethal); and 6 = 38° and above (high lethal). Colours represent different loggers from the same site.

**Figure 4:**
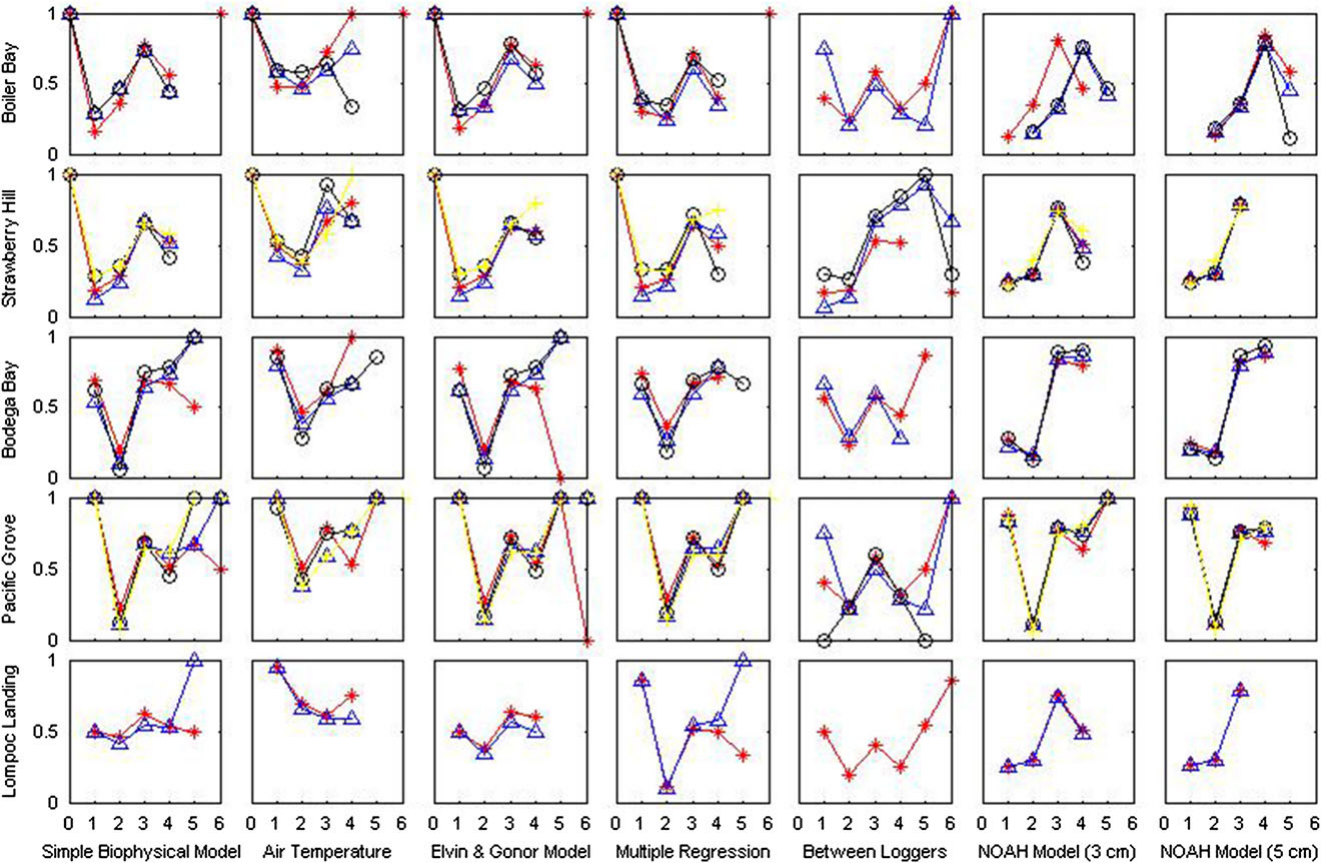
False-alarm ratios for all models at all sites. Category labels are as in Fig. 3.

Peirce, Heidke and Gerrity scores indicated relatively little difference between any of the modelling approaches examined, although on average the 3 cm layer of the NOAH model tended to have the highest skill scores (Table 3). However, when applied to a physiological framework, each predictive method demonstrated different levels of skill at different points along the physiological performance curve, and the temperatures at which each model displayed the highest skill varied with geographical location. At all sites, hit rates indicated more success (values closer to 1) at low to mid temperatures than at high temperatures, with the highest values being reported for the 10–17°C range at most sites (Fig. 3). In contrast, false-alarm ratios for the lowest temperature category were also high at all sites, indicating the tendency of all models to ‘run cold’ (Fig. 4), as also seen by the negative values for bias (Table 2).

Results from the interlogger analysis indicated almost-perfect hit rates at all sites for temperatures falling between 10 and 17°C (low sub-optimal). Overall, hit rates tended to decrease as temperatures increased at all sites, although at Boiler Bay, Strawberry Hill and Pacific Grove, hit rates for the 22–32°C category (high sub-optimal) were higher than those for 17–22°C (optimal). At four sites, hit rates decreased to 0 for temperatures above 38°C (high lethal); the notable exception is Strawberry Hill, for which interlogger hit rates indicated that loggers were recording similar high temperatures. This again may reflect the observation that at this site, which has a broad intertidal bench, summer-time low tides occur midday (Helmuth *et al*., 2002), so that variability in temperature among loggers created by cooling from unaccounted for wave splash was likely to be low.

False-alarm ratios for the interlogger analysis varied across categories and across sites (Fig. 4). Owing to the nature of forecasts with multiple categories, a ‘miss’ in one category is a ‘false alarm’ in another. As a result of the high variability in maximal temperatures and small sample sizes for the two highest categories (32–38 and >38°C), the false-alarm ratio was at times undefined owing to division by zero. Frequently, false-alarm ratios for the interlogger analysis had values closer to 1 (indicating less skill) at high temperatures, although this trend was not consistent across sites.

At all sites, hit rates based on air temperature were highest for the low sub-optimal band of 10–17°C; at Bodega Bay and Lompoc Landing, low sub-optimal hit rates exceeded 0.9. However, hit rates for the next highest temperature category (optimal, 17–22°C) were quite poor, and virtually no skill was gained by using air temperature as a proxy for body temperature within the sub-optimal high, sub-lethal high and lethal high categories. These results again strongly caution against the use of air temperature as a direct metric for heat stress except possibly for extreme cold, because, at all sites, air temperature greatly under-predicted the number of instances mussels experienced optimal, high sub-optimal, high sub-lethal and high lethal temperatures.

Hit rates for the Elvin and Gonor model were generally comparable to or slightly lower than hit rates for air temperature alone at the low sub-lethal and low sub-optimal levels, but indicated more model skill in predicting temperatures at physiologically optimal levels. Although the Elvin and Gonor model was developed for a site near Strawberry Hill and Boiler Bay, the hit rate was relatively low for high sub-optimal temperatures at these sites. Hit rates indicated some skill at predicting high sub-optimal temperatures at Bodega Bay and Pacific Grove (Fig. 3).

The skill of multiple regression approaches varied from site to site and from category to category. At Strawberry Hill, Bodega Bay and Pacific Grove, hit rates for multiple regression approaches were similar to those of the Elvin and Gonor model, whereas at Lompoc Landing and Boiler Bay, hit rates indicated more skill at predicting temperatures at high sub-optimal levels.

Hit rates for the steady-state biophysical model were generally lower than other approaches at low sub-lethal and low sub-optimal temperatures, but this model exhibited higher hit rates than other methods for the 22–32°C (high sub-optimal) range at Bodega Bay and Pacific Grove.

Hit rates for the time-dynamic NOAH mussel biophysical model were comparable to those of the steady-state biophysical model but higher than other approaches for the 22–32°C (high sub-optimal) range (Fig. 3). False-alarm ratios tended to be lower at cooler temperatures than other approaches (Fig. 4).

A common trend among all models were hit rates of 0 and incalculable false-alarm ratios for temperatures >38°C, which indicates that although loggers reached these high temperatures, these conditions were not predicted by the models. Results also indicate a lack of model stationarity across some sites, most notably between Lompoc Landing and the other sites. Here, recorded temperatures exceeded temperatures predicted by the steady-state and time-dynamic biophysical models (Fig. 4), and in terms of both physiological bins and overall statistics, the most ‘skilled’ model at higher temperatures was the multiple regression tailored specifically for that site. The failure of the models at Lompoc Landing may indicate gaps in our ability to account for the effect of local topography. Given that the more complex biophysical models have been observed to perform well at certain sites and less well at others (Tables 2 and 3; and Gilman *et al*., 2006b; Wethey *et al*., 2011a), this suggests that a more thorough understanding of the effect of topography on the interactions between environment and organism is necessary to apply the assumption of model stationarity to different locations in space (Denny *et al*., 2011).

## Discussion

Surface air temperatures are predicted generally to increase under future climate scenarios (IPCC, 2013), but understanding how increases in air temperature will likely affect ectothermic organisms can be difficult to assess given that air temperature is only one component of an organism's thermal energy budget. Multiple models have shown that all other factors (e.g. solar radiation, wind speed) being equal, increases in air temperature will generally lead to concomitant increases in body temperature (Gilman *et al*., 2006b) and, thus, will shift organisms’ positions along their physiological performance curves to the right (Fig. 1). The physiological and ecological consequences of such a shift, however, are strongly determined by the baseline temperature in relation to the organism's thermal optimum. As a result of the left-skewed nature of physiological performance curves, even a slight increase in body temperature could significantly affect physiological performance when temperatures are already near or above optimum. Although ectotherms are able to acclimatize to local environmental conditions to some degree, it is virtually certain that physiological performance will vary at least in terms of interpopulation variation along a species range and even among individuals in the same population (Dowd *et al*., 2015). It is thus imperative that we develop methods for estimating stress levels over large geographical ranges that account for not only variability in environmental drivers but also variability in physiological vulnerability among individuals (e.g. Mislan *et al*., 2014; Kingsolver and Woods, 2016). Moreover, not only are air temperatures anticipated to change, but so too are wind patterns as well as cloud cover and, thus, solar radiation, which will confound any effects of changes in air temperature on animal body temperature (Helmuth *et al*., 2011).

It is important to be able to predict sub-lethal levels of stress as well as lethal conditions, and models designed to make these predictions must perform well along the entire width of the physiological performance curve (Woodin *et al*., 2013). Our results suggest that it may be necessary to develop multiple models, each of which is ‘tuned’ to predict specific outputs, such as the incidence of lethal extremes or time spent at sub-optimal temperatures, depending on the application to which the model is being applied. For example, the simplified Helmuth *et al*. (2011) steady-state biophysical model was designed to capture instances when mussels experienced high, sub-lethal temperatures, as reflected by higher hit rates in the high sub-optimal range than other approaches, especially when applied to sites geographically near the site at which it was developed. However, this tuning is likely to have reduced the effectiveness of the model to capture low sub-lethal stress and, possibly, high lethal temperatures. This shortcoming was probably exacerbated by our focus on daily maximal rather than daily minimal temperatures. Model tuning, therefore, will be likely to require advanced understanding of what categories of performance are most important to predict, and must recognize that tuning for one performance category may involve trade-offs in the ability to predict others accurately.

To a great extent, what suffices as ‘good enough’ in terms of model accuracy will depend on the application to which the results are applied. If, for example, the primary parameter of interest is the risk of mortality from exposure to extreme high temperatures, then all of the models used here would fail unless the threshold of mortality is reduced (e.g. to 36°C) in order to lower the chance of false negatives. If the prediction of overall levels of stress in the absence of mortality is the primary forecasting goal, for example as a means of predicting heat shock protein production (e.g. Place *et al*., 2008), then either of the biophysics models would be likely to provide sufficient information. Once tuned (a common modelling exercise), the regression models too would likely have some predictive power, at least within the range of environmental parameters used to tune the model (Olsen *et al*., 2016). Even in these cases, however, tuning would be likely to involve trade-offs between predicting high and low temperatures.

Probability distributions of body temperature are often predicted to be centred slightly below optimal because under natural selection this maximizes overall fitness during fluctuating conditions when ‘cold’ deviations are less physiologically stressful than ‘hot’ deviations (Martin and Huey, 2008). Individuals that select microhabitats of this type may reduce the probability of experiencing lethal extremes (Gaines and Denny, 1993; Woodin *et al*., 2013). Whether such generalizations hold true for a wide diversity of organisms, and whether they apply over a species’ entire geographical range, is unclear. However, in light of these ideas, the tendency of model performance to decrease at higher temperatures is especially troubling. The fact that, in many cases, hit rates were 0 (and false-alarm ratios incalculable owing to division by 0) for the sub-lethal high and lethal high categories indicate that the particular models used here may be ineffective predictors of lethally high temperatures unless they are applied to an adjusted threshold. However, it should be noted that the loggers themselves have errors of ~2°C in their measurements (Fitzhenry *et al*., 2004) and that there is high variability among mussels/loggers at any site (Denny *et al*., 2011; and Table 2). If all mussels living on horizontal substrata at a site truly had experienced temperatures exceeding 38°C, this should have caused mass mortality (Harley, 2008; Mislan *et al*., 2014), which was not observed. We also tested model skill in forecasting stress levels on a particular day. Previous tests of model skill in predicting temperature have been higher when, for example, the metric being tested is monthly maximal temperature (Gilman *et al*., 2006b). If one cares less about stress on a given day and more about the cumulative probability over a longer time period, then observed model skill might have been higher. Regardless, this result points to difficulties inherent in forecasting rare, extreme events, and suggests that consideration be given to the relative importance of false positives and false negatives (see below). Notably, however, although biophysical models were imperfect, they were far more accurate than simply using air temperature.

Extreme events are also rare events (Casati *et al*., 2008; IPCC, 2012), and this rarity leads to small sample sizes and, as a result, large sampling uncertainties. For example, although several years of data were pooled at each site in this study, sample sizes of observed temperatures for the high sub-lethal and high lethal categories consisted of only a handful of data points. However, high sub-optimal temperatures are not rare occurrences, and at the two central sites (Bodega Bay and Pacific Grove), hit rates for the biophysical models for high sub-optimal temperatures were higher than those for other methods, further emphasizing the importance of a physiological basis to model verification. These results are consistent with recent modelling that suggests that local and geographical limits may in some cases be determined more by chronic exposure to sub-lethal temperatures rather than to lethal events (Sará *et al*., 2011; Mislan *et al*., 2014; Mislan and Wethey, 2015). This arises because mortality is influenced by both the magnitude and the duration of thermal stress at temperatures below the absolute lethal limit (Foster, 1969).

Both cold stress and heat stress events are time dependent, and whereas short-term exposures to extremes can result in the restructuring of an ecosystem (Wethey *et al*., 2011b), sub-lethal effects can lead to reproductive failures (Petes *et al*., 2008) and negative energy balance (Fly and Hilbish, 2013; Fly *et al*., 2015). Moderate temperatures may become lethal as the window of exposure is expanded. For example, although *M. californianus* was shown to survive short-term (8 h) exposures to extremely cold temperatures (2°C), as of yet, it is unknown how chronic or long-term exposures to these temperatures may affect physiology (Yao and Somero, 2012). Given that these chronic exposures to sub-lethal temperatures can be as ecologically relevant as short-term extreme events, any models designed to predict the effects of climate change on organism performance and survival need to reflect sub-lethal temperatures accurately as well as lethal temperatures (for examples, see Jones *et al*., 2010; Mislan *et al*., 2014; Mislan and Wethey, 2015). Testing model skill using metrics that incorporate both the magnitude and duration of exposure to temperatures above some fixed threshold would be valuable, akin to the use of degree heating weeks for corals (van Hooidonk and Huber, 2009) and growing degree days for plants (Sykes *et al*., 1996). However, although lethal temperatures of *M. californianus* are known to vary with exposure duration (Mislan *et al*., 2014), insufficient data exist to apply a degree heating metric.

When making forecasts that have societal implications, an effort is often made to reduce false positives when making predictions of the effects of climate change, because ‘crying wolf’ erodes public confidence and trust (Anderegg and Goldsmith, 2014). However, false negatives impair communication of risk and can prevent implementation of appropriate adaptation and mitigation strategies, for example the protection of vulnerable species and populations (Selkoe *et al*., 2015). As such, it is important to assess the relative implications of false positives and false negatives, because this can elucidate the relative importance of the two types of model failure. False-alarm ratios in this study indicated that models were more likely to predict temperatures cooler than what mussels genuinely experienced; in essence, predictions were overly conservative. As a result, mitigation efforts that use models like these would do well to account for the fact that mussels will be likely to experience warmer temperatures more frequently than predicted in this study. As described above, the addition of a ‘buffer’ (e.g. by lowering lethal thresholds by a few degrees) would make these models less conservative; the tolerance for false positives and false negatives would then define the magnitude of this buffer.

It is important to note that three of the methods used here are based only on one or two environmental parameters, and that even the biophysical models used in this study make a number of simplifying assumptions in order to facilitate accessibility to a range of users (Helmuth *et al*., 2011; Wethey *et al*., 2011a). In addition, for rocky intertidal organisms, grounding a model in physiology may be complicated by spatial heterogeneity of the intertidal zone itself (Denny *et al*., 2011). In this study, the use of data from one logger as a forecast for another also indicated low levels of skill at high temperatures, even though data were collected from microsites with similar wave exposures and substratum angles, further emphasizing how localized heat stress events may be (Tables 2 and 3). This point is important because it indicates inherent difficulty in predicting the incidence of lethality in any environment where there is high spatial and temporal heterogeneity in temperatures (Potter *et al*., 2013). Denny *et al*. (2011), for example, found that while the mean range of maximal body temperatures among individual mussels in a single exposed bed was 3.7°C, inter-individual variation in body temperature could be as large as 11.7°C. We also found large variations among loggers (Table 2), with maximal inter-individual variation of 17°C at Boiler Bay, 22.6°C at Strawberry Hill, 22.3°C at Bodega, 18.2°C at Pacific Grove and 12.6°C at Lompoc. This occurred even though all loggers were deployed on substrata with similar exposures to the sun, and is likely to be a result of unaccounted for differences in wave splash among microsites, which was not taken into account. As a result, even the most skilled models available may not be able to predict explicitly the real temperatures that all organisms in the field will experience at any given moment but rather may provide estimates of the probability that certain thresholds of stress will be reached. Higher skill is also likely when temperatures are considered over longer time scales. For example, the maximal differences listed above represent points in time when one logger was splashed by a wave but another was not. The average difference among loggers was 2–3°C (Table 2), very similar to the error in the instruments themselves (Fitzhenry *et al*., 2004).

Despite the inter-individual variability in predicting high body temperatures and uncertainty in verifying predictions in this region, this analysis provides some insight into the methods necessary to gain an understanding of physiological stress in the field. The ability of air temperature to serve as an effective proxy for body temperature decreases sharply once body temperatures reach or rise above what could be considered ‘optimal’ (17–22°C). Although a variety of factors influence the thermal regime of rocky-shore organisms, high temperatures are driven primarily by interactions of air temperature (a climatic factor) and solar radiation (a non-climatic factor; Southward, 1958; Denny and Harley, 2006; Marshall *et al*., 2010; Mislan *et al*., 2014). Solar radiation, in particular, has a profound effect on the maximal temperatures experienced by *M. californianus* (Helmuth, 1998), reflected in the poor predictive capacity of air temperature with regard to physiological heat stress. Recent studies have suggested that thermal adaptation of certain intertidal species may be driven primarily by adaptation to non-climatic sources of heat (Marshall *et al*., 2010); however, most studies do not distinguish between heating from climatic (ambient air temperature) and non-climatic (solar heating) sources (Helmuth *et al*., 2006). Climate change could indirectly affect the amount of solar irradiance reaching the surface through changes in cloud cover. Thus, a thorough understanding of non-climatic sources of heat and their interaction with climatic sources will be necessary to gain an understanding of how climate change might affect intertidal systems. As a result, even though the models tested here exhibited error, all were substantially more accurate than simply using air temperature as a proxy.

### Conclusions

Our results highlight the need for a better understanding of how large-scale climatic shifts drive patterns of local weather that ultimately manifest as changes in physiological performance. Although simple environmental metrics, such as air or surface temperature, may provide a rough idea of how organisms will respond to climate change, they need to be considered in the same way as climate indices such as the El Niño Southern Oscillation and North Atlantic Oscillation (Stenseth *et al*., 2003). That is, air temperatures (and especially average air temperatures) are diagnostic only to the extent that they covary with aspects of the environmental signal that drive organismal biology (Hallett *et al*., 2004). As a result of the inherent stochasticity in body temperature drivers and high variability of the drivers themselves, there may be a limit to the accuracy of body temperature predictions, especially in habitats with high levels of topographic heterogeneity. Notably, however, this is not simply an issue with the method used to model body temperatures *per se*. Indeed, direct measurements of body temperature in the field would present similar challenges (Denny *et al*., 2011), as would field measurements of physiological state (Dowd *et al*., 2015). This argues that in many habitats the average temperature (or metrics of physiological state) of organisms may be far less meaningful than frequency distributions that account for the effects of both microhabitat heterogeneity (Potter *et al*., 2013) and inter-individual variability in thermal sensitivity (Dowd *et al*., 2015). Methods that account for this variance are sorely needed (Denny *et al*., 2011). Regardless, the results of the study presented here suggest strongly that tests of model skill based on physiology are much more likely to give realistic estimates of the ability of a model to capture patterns of biological response than simple metrics, such as air or water temperature, or measurements of accuracy based only on body temperature. They also provide guidance for how models should be tuned for specific applications. For example, an error of only a few degrees at temperatures above the physiological optimum may have more serious consequences than an error of the same magnitude at temperatures below optimum; as a result, current models may be better equipped to predict sub-lethal rather than lethal effects. The quest for predictive methods that can assist decision-making in the face of rapid climate change has gained prominence in recent years. It is also becoming increasingly clear that simply relying on generalizations and trends while ignoring the often overwhelming importance of spatial and temporal variability in environmental conditions and the response of organisms to those conditions is likely to lead to some very unfortunate surprises. Although difficult, the inclusion of physiological information in mechanistic forecasts is a crucial step in moving towards this goal.
